# Clinical Potential of Fruit in Bladder Cancer Prevention and Treatment

**DOI:** 10.3390/nu14061132

**Published:** 2022-03-08

**Authors:** Paulina Wigner, Michał Bijak, Joanna Saluk-Bijak

**Affiliations:** 1 Department of General Biochemistry, Faculty of Biology and Environmental Protection, University of Lodz, Pomorska 141/143, 90-136 Lodz, Poland; joanna.saluk@biol.uni.lodz.pl; 2 Biohazard Prevention Centre, Faculty of Biology and Environmental Protection, University of Lodz, Pomorska 141/143, 90-136 Lodz, Poland; michal.bijak@biol.uni.lodz.pl

**Keywords:** bladder cancer, prevention, apple, cactus pear, citrus, pomegranate, cranberry

## Abstract

Bladder cancer (BC) is the most common tumor of the urinary system in the world. Moreover, despite using anticancer therapies, BC is also characterized by a high recurrence risk. Among numerous risk factors, cigarette smoking, occupational exposure to certain aromatic compounds, and genetic factors contribute most strongly to BC development. However, the epidemiological data to date suggests that diet quality may influence some carcinogenic factors of BC and, therefore, might have a preventative effect. Adequate consumption of selected fruits with scientifically proven properties, including pomegranates and cranberries, can significantly reduce the risk of developing BC, even in those at risk. Therefore, in this article, we aim to elucidate, using available literature, the role of fruits, including pomegranates, cranberries, citrus fruits, cactus pears, and apples, in BC prevention and treatment. Previous data indicate the role of compounds in the above-mentioned fruits in the modulation of the signaling pathways, including cell proliferation, cell growth, cell survival, and cell death.

## 1. Introduction

Bladder cancer (BC) is the most common tumor of the urinary system in the world. On the list of the most common types of cancer, BC ranks in fourth place among men, while in the female population, BC is in eighth place [1]. According to GLOBOCAN data, more than 550,000 new BC cases and 200,000 BC deaths have been reported worldwide each year. The highest incidence rates were recorded in the countries of southern and western Europe, as well as in North America. Among the analyzed countries, the highest incidence is in Lebanon (25/100,000) and Greece (21.2/100,000) and the lowest-in China (3.7/100,000) and Brazil (5/100,000). Similarly, these countries are characterized by the highest (Lebanon 9.3/100,000 and Greece (4/100,000) and the lowest (China 1.6/100,000 and Brazil 1.7/100,000) mortality rate. By comparison, in the United States, the BC incidence rate is 12/100,000, while the mortality rate is 2.1/100,000 [2].

The most significant BC risk factors include cigarette smoking, occupational exposure to certain aromatic compounds, and genetic factors [3,4,5]. Moreover, the epidemiological data suggest that diet quality may influence some carcinogenic factors of BC and, therefore, can prevent or accelerate the development of cancer [6]. A significant positive association between the Western diet and BC development confirms the role of diet in BC etiology. Data from 13 prospective cohort studies in the *Bladder Cancer Epidemiology and Nutritional Determinants* (BLEND) study has shown that the Western diet doubles the risk of BC development [7]. The Western dietary pattern is based on high consumption of red and processed meat, fast food, finished products, sweet soft drinks, eggs, refined cereals, snacks, high-fat dairy products, and hydrogenated fats. On the other hand, there is evidence that the Nordic diet positively affects human health [8]. The Nordic diet includes unhealthy foods, such as red meat, sugar, and full-fat dairy products, and six foods (fish, whole grain bread, cabbage, barley, apples and pears, root vegetables) with health benefits [9]. Moreover, numerous studies have shown the beneficial effect of traditional Nordic healthy food products on preventing BC [10,11].

BC is characterized by a high risk of relapse, accompanied by relatively rapid progression. Competitive risk regression analysis demonstrated that the 2-year, 5-year, and 10-year BC recurrence rates were 61.1%, 69.5%, and 74.3%, respectively [12]. Moreover, in more than 50% of BC cases, the patient reports muscle invasion, followed by metastases to lung tissues and the liver [13]. Metastases to diverse organs contribute to a poor prognosis, and the 5-year survival rate of patients with BC is below 60% [13]. Therefore, the diagnosis and treatment of BC are associated with constant monitoring and repeated clinical interventions after surgery, making BC treatment far costlier than any other cancer [14]. However, despite significant advances in conventional treatment techniques, the prognosis for BC patients is very poor, and the mortality rate is very high [15]. Conventional BC therapy includes chemotherapy, radiotherapy, and surgery [16]; however, currently, the main obstacle in chemotherapy is the high resistance and low sensitivity of BC [17]. Unfortunately, in preclinical studies, many compounds showing effective anticancer properties in fighting BC have proven ineffective in clinical trials [17]. Therefore, discovering new therapeutic agents for the treatment and prevention of BC is urgently needed.

Recently, a series of studies have been carried out on the effects of natural products on preventing many types of cancers [18,19]. The *2010 Dietary Guidelines for Americans* encourage increased fruit intake as part of a healthy dietary pattern [20]. In turn, the World Health Organization (WHO) recommends 400 g (equivalent to five portions) of fruits and vegetables in a daily diet. According to WHO data, fruit should include fresh, frozen, cooked, canned, or dried fruits, excluding fruit juices and salted or pickled fruits. In turn, vegetables in a diet should include fresh, frozen, cooked, canned, or dried vegetables, excluding legumes and salted or pickled vegetables, juices, nuts, seeds, and starchy vegetables such as potatoes or corn. Such consumption of fruits and vegetables prevents the development of non-communicable diseases (including diabetes, heart disease, strokes, and cancer) and prevents and mitigates the deficiencies of several micronutrients. Epidemiological data to date has shown that insufficient consumption of fruit and vegetables causes 14% of deaths from gastrointestinal cancer worldwide, about 11% from ischemic heart disease, and about 9% of deaths from strokes [21,22]. Previous epidemiological studies showed an inverse correlation between fruit/vegetable consumption and BC incidence [18,19]. The significant role of diet in the combat of urinary bladder carcinogenesis is feasible since most compounds and their metabolite products are excreted with urine. Thus, it directly interacts with urothelium cells. Moreover, the concentration of metabolically active compounds is higher in urine than other body fluids and tissues, thereby including cancer-preventive components, which may provide desirable anticancer health. Anticancer food ingredients such as phenols, flavonoids, ellagitannins, tannins, and proanthocyanidin are contained in the highest concentration in apples, pomegranates, citrus fruits, cranberry, and cactus pears. A sufficiently high supply of these natural compounds may turn out to be effective in the prevention of BC. Therefore, in this publication, we want to clarify, using the available literature, the role of fruits, including pomegranates, cranberry, citrus fruits, cactus pears, and apples in anti-BC defense.

## 2. Pomegranate

### 2.1. Pomegranate—Basic Information

Pomegranate (*Punica granatum* L.) is an edible fruit widely grown in Mediterranean countries, northern India, northern/southern America, Europe, and even in Taiwan, mainly as gardening trees [23]. Pomegranate fruits consist of white to dark purple edible seeds embedded in a white spongy astringent membrane surrounded by a thick reddish skin or pericarp. Peel makes up almost 50% of the fruit weight and it is a rich source of bioactive ingredients, including phenols, ellagitannins, flavonoids and proanthocyanidins. It also comprises several minerals, mainly potassium (K), calcium (Ca), phosphorus (P), magnesium (Mg), sodium (Na), and nitrogen (N), as well as complex polysaccharides [24,25]. The remaining 50% of pomegranates are seeds (constituting 10% of the mass of the fruit) and warp (constituting 40% of fruit mass) [24]. Pomegranates are characterized by strong anti-inflammatory and antioxidant properties due to the high content of hydrolyzable tannins (munialin, pedunculagin, yanidin-3-glucoside) [26,27]. Various organic acids, including citric acid, ascorbic acid, and malic acid, are also present in the seed coat [24], while matrices contain water, pectin, and sugars, mainly fructose and glucose. Pomegranate leaves contain several unique tannins, in addition to apigenin glycosides, i.e., flavone with progestogenic and anxiolytic properties [28,29]. Leaves are also a rich source of elements such as N, K, Ca, and iron (Fe), the levels of which vary depending on the season, stage, and maturity of the plant [30]. For example, it has been reported that the K content is high in young leaves, while Ca and Fe levels are the highest in old ones. Thanks to this richness of various compounds, the pomegranate exhibits pro-health action, including anticancer properties by inhibition of cell proliferation, cell cycle arrest, and activation of apoptosis [30,31]. Since ancient times, the pomegranate has been used to treat diarrhea [32], parasitic infections [33], and diabetes [34]. Previous results confirm that various parts of this plant, such as the peel, flower, and seeds, have antidiabetic properties. Although the mechanism of the ability of pomegranate extracts to lower blood glucose is not known, the obtained results suggest the role of pomegranate seeds and flower extracts in an increased peripheral glucose utilization inhibition of the proximal tubular reabsorption mechanism for glucose in kidneys [34]. Greco-Arab and Islamic medicine have prescribed the pomegranate for sore throats, inflammation, and rheumatism [35]. Due to the high content of triacylglycerols and lignin in pomegranate seeds, the oil obtained from them has antioxidant properties and, therefore, has been used to alleviate inflammation. Similarly, pomegranate juice has a strong anti-inflammatory effect due to the abundance of compounds such as tannins and flavonoids in folk medicine; after boiling and drying them, pomegranate peels have been used in the local treatment of aphthas as well as in the treatment of diarrhea [35]. Pomegranate’s multifaceted activities (anti-inflammatory, antioxidant, and anticancer) have prompted more and more research into its use in solving many medical problems [30]. Previous studies confirmed that pomegranate juice might be used in prostate cancer prevention. Men with a high level of prostate-specific antigen (PSA) following surgery or radiation, after using a pomegranate juice-supplemented diet, were characterized by decreased PSA concentration compared with men with a non-supplemented diet. Animal studies also confirm the anticancer properties of pomegranate peel and seeds [30].

Any substance introduced into the body can cause side effects. However, in the case of pomegranate ingredients, in vitro and animal studies did not confirm any adverse effects at the doses tested. Consequently, in a study of 86 patients who received 1420 mg/day of pomegranate extract tablets for 28 days, no side effects or adverse changes in the urine or blood of the study participants were reported [36,37]. However, the consumption of pomegranate may affect the oral bioavailability of drugs [38]. The analysis showed an inhibitory effect of pomegranate juice on CYP2CP in liver cells (a gene that codes for an enzyme that breaks down warfarin in the body) and an increased bioavailability of tolbutamide (a CYP2CP substrate). In addition, it has been suggested that pomegranate may inhibit the cytochrome P450-3A4 (CYP3A4)-mediated metabolism of carbamazepine. [36,39,40]. CYP2CP and CYP3A4 belong to the group of enzymes that function as monooxygenases. These enzymes oxidize various compounds, including steroids, fatty acids, and xenobiotics (e.g., drugs), and thus play an important role in the clearance of toxicity compounds [36,39,40].

### 2.2. Pomegranate in Bladder Cancer Prevention

Most of the results to date confirming the anticancer effect of pomegranates have been obtained in in vitro studies. These studies focus primarily on identifying the molecular mechanisms of the anticancer activity of pomegranate ingredients, including angiogenesis, apoptosis, and cell cycle arrest. Moreover, due to their various compositions, the individual components of the fruit may differ in the action specificity. Chang et al. found that the ethanol extract of pomegranate peel exhibited better inhibitory activity to the growth of human urinary bladder urothelial carcinoma T24 and J82 cells than the pulp extract [23]. Moreover, among studied fractions, the extract from pomegranate peels, labeled as the PEPE2 fraction, showed the highest inhibitory activity against urinary bladder urothelial carcinoma cells along with the least influence on normal-like urothelial E7 cells. This fraction was also attributed to the apoptosis of the urinary bladder urothelial carcinoma cell-mediated by death receptors, mitochondria, and endoplasmic reticulum. Furthermore, the result of the in vitro study had been confirmed in an in vivo study. The xenograft-induced bladder tumor in nude mice demonstrated that the oral consumption of the PEPE2 fraction (2, 5, 10, and 100 mg/kg) could decrease the volume and weight of T24 tumors and cause cell apoptosis in the xenografted tumors [23]. Similarly, Lee et al. showed that the edible portion of Taiwanese pomegranate fruit ethanol extract effectively inhibited the proliferation and induced apoptosis of urinary bladder urothelial carcinoma cells (lines T24 and J82) [41]. Moreover, the cell cycle analysis reported that the studied pomegranate ethanol extract might cause an increase in the cyclin A protein level and reduce the cyclin-dependent kinase 1 expression, finally leading to S phase arrest [41]. Moreover, the treatment of urinary bladder urothelial carcinoma cells (T24 and J82 cell lines) with pomegranate extract increased the expression of profilin 1 to increase the expression of the PTEN (phosphatase and tensin homolog) gene, which, in turn, may inhibit the Akt/mTORC1 (phosphoinositide 3-kinase/mammalian target of rapamycin complex 1) signaling pathway to prevent the proliferation/migration of urothelial BC cells. PTEN protein dephosphorylates phosphatidylinositol-3,4,5-triphosphate. This dephosphorylation inhibits the activity of the Akt protein and, thus, the Akt signaling pathway, which plays a key role in protein synthesis, metabolism, and cell proliferation [42]. While the active form of Akt can phosphorylate tuberin/TSC2 and thus induce the mTORC1 complex, mTORC1 can promote cell growth under favorable conditions or catabolic processes under unfavorable conditions [43]. Blocking the mTORC1 pathway prevents the synthesis of proteins incompatible with the progression of the cell cycle, which additionally enhances the cell cycle arrest.

A subsequent in vitro study showed that pomegranate ethanol extract treatment could restrict urinary bladder urothelial carcinoma cell (lines T24 and J82) proliferation and migration. The proteomic analysis detected the de-regulated expression of 20 proteins involved in apoptosis (BCL10, Diablo, eIF5A, NDUFAF1, UBQLN1, XIAP, and BAG2), cell proliferation (PSMF1, PFN1 (pefin), TPD52L2), DNA metabolism (UBQLN1), and proteasome (UBQLN1, PSMD9, PSMF1). Among studied proteins, levels of pro-apoptotic proteins were increased after pomegranate extract treatment of cells in both T24 and J82 lines. BCL10 is an apoptotic regulatory protein involved in the Apaf1/caspase 9-mediated cell death pathway [44]. eIF5A (eukaryotic translation initiation factor 5A) is a pro-apoptotic protein occurring exclusively during apoptosis [45]. In turn, TPD52L2 is involved in signal-regulating kinase 1 (ASK1)-induced apoptosis [46]. A PFN1 (a Ca^2+^-binding protein that belongs to the penta-EF hand protein family) can regulate Fas-executed apoptosis [47]. The apoptotic inhibitory function of XIAP (X-linked inhibitor of apoptosis protein) can be abolished by Smac/Diablo and Omi/HtrA2 proteins released from mitochondria along with cytochrome c (Cyt c). BAG2 (BCL2-associated athanogene 2) is also a pro-apoptotic protein that is upregulated in proteasome inhibitor-induced apoptosis [48]. Moreover, pomegranate ethanol extract may modify the expressions of several genes associated with 26S proteasome action (*PSMD9*, *UBQLN1*, *PSMF1*). The high proteasomal activity is important in carcinogenesis since it may contribute to tumorigenesis by antiapoptotic protection and result in a survival advantage [49]. Pomegranate extract increased the expression of *PSMF1/PI31* while reducing the expression of *NF-κB* (nuclear factor kappa-light-chain-enhancer of activated B-cells) in vitro. PSMF1/PI31 can bind to the 20S catalytic molecule of the 26S proteasome, making it difficult for the substrate to access the enzyme core and thus inhibiting the activity of the proteasomes [50]. Suppression of proteasome activity may prevent the degradation of IκB (endogenous NF-κB inhibitor) and the subsequent activation/translocation of NF-κB into the nucleus [51]. NF-κB plays an important role in the oncogenesis of many types of cancers by promoting cell proliferation, migration, and suppression of apoptosis [52]. Therefore, the effect of pomegranate extract on NF-κB inhibits cell proliferation and migration and contributes to the induction of apoptosis, preventing the development and progression of BC. To sum up, the undoubted advantage of pomegranate extract is its ability to induce apoptosis through multiple pathways. As a result, the end signal of apoptosis activation is strongly amplified.

Interestingly, an in vitro study indicated that exposure of BC cells to the edible portion of pomegranate ethanol extract might result in impaired aerobic glycolysis via the induction of the *NDUFAF1* expression increase [53]. NDUFAF1 is a chaperone involved in the construction of the mitochondrial NADH complex: ubiquinone oxidoreductase (complex I), which transfers an electron from NADH to ubiquinone (coenzyme Q) in the first stage of the mitochondrial respiratory chain [54]. Cancer cells multiply very quickly and depend on high metabolic activity. To meet their high energy requirements, cancer cells use oxygen glycolysis to acquire energy from glucose (Warburg effect). Increasing *NDUFAF1* expression blocks the efficiency of glycolysis and thus reduces energy production, thereby inducing apoptosis of cancer cells [55].

The anticancer properties of pomegranate have also been confirmed by Sun and colleagues [56]. A 72-hour exposure to the extract from dried *Punica granatum* plant material decreased the viabilities of HT-1197 and RT4 BC cells in a concentration-based manner. It also efficiently inhibited the growth of colonies compared to untreated cells. Moreover, the studied extract caused decreased miR-10b expression in both HT-1197 and RT4 cells [56]. miR-10b showed an oncogenic role in several types of cancers, including breast, gastric, colorectal, and laryngeal cancers [57]. Moreover, in the cancer course, including glioma, colon, ovary, and gastric cancer, it has been observed that there is a decreased expression of *HOXD10*, which has been identified as a target for miR-10b. In turn, *Punica granatum* extract exposure (dried *Punica granatum* plant material) of HT-1197 and RT4 cells reduced miR-10b expression and consequently increased HOXD10 protein and mRNA levels. Therefore, the exposition of *Punica granatum* extract suppressed migration ability relative to the control of extract-untreated cells [56]. Some results also suggest that the upregulation of miR-10b expression elevates the migration potential and invasive properties of bladder carcinoma cells [58]; thus, pomegranate extract treatment has the potential to inhibit the development and progression of BC.

As mentioned above, edible seeds are not the only sources of health-promoting compounds. Zeng and colleagues showed that the main compounds of pomegranate peel, characterized by good anticancer activity, were tannins [59]. The key tannin of the extract obtained was gallic acid. An in vitro study confirmed that gallic acid showed significantly higher inhibition of T24 cell viability among pomegranate peel tannins than the other three monomers (punicalagin, punicalin, ellagic acid). Moreover, a comparative analysis of the effectiveness of gallic acid and 5-fluorouracil showed that gallic acid significantly reduced cell viability, especially after 24 and 48 hours of exposure [59]. 5-fluorouracil is a common anticancer drug that can interfere with DNA synthesis by acting primarily on the S phase of the cell cycle and is also a chemotherapeutic drug for bladder irrigation after surgery [60]. In turn, analyses of gallic acid mechanism action showed that this compound influenced the morphology of T24 cells, inhibited cell proliferation, blocked the S-phase cell cycle, and induced cell apoptosis, mainly in the early stages [59]. Subsequent studies confirmed Zeng’s results that T24 cells, after exposure to gallic acid, were characterized by elevated mitochondrial ROS (reactive oxygen species) levels and lowered mitochondrial membrane potential. Moreover, after treatment with gallic acid, the expression level of Bax (BCL2-associated X protein), p53, caspase-3, and Cyt c proteins, as well as genes, was significantly increased. At the same time, BCL2 (B-cell CLL/lymphoma 2) significantly decreased. The p53 protein can increase the expression of the pro-apoptotic protein Bax and decrease the expression of the antiapoptotic protein BCL2 and trigger apoptosis via the mitochondrial pathway [61]. In turn, Cyt c is often released from mitochondria during the early stages of apoptosis and can thus enhance apoptosis signaling as well as directly regulate apoptosis. Caspase-3, called the death protease, is an effector caspase, which can lead to chromatin shrinkage, DNA fragmentation, cell lysis, and, finally, cell death [62]. Thus, these results confirmed that the observed decrease in the cell viability of BC cells after gallic acid exposure might be a consequence of the induction of mitochondria-dependent apoptosis.

On the other hand, the ability to induce apoptosis is not the only antitumor property of gallic acid obtained from pomegranates. Tests assessing the ability to migrate and invade cells showed that gallic acid significantly inhibited the migration and invasion of T24 cells, accompanied by decreased expression of intracellular VEGF (vascular endothelial growth factor) protein and lowered secretion of extracellular VEGF [59]. VEGF is the main factor in stimulating tumor angiogenesis, which can promote the growth of vascular endothelial cells and induce vascular proliferation, which is closely related to tumor progression. Essential studies have shown that VEGF is linked to metastatic activity and can induce angiogenesis and promote cancer cell metastasis [63,64]. Therefore, reducing VEGF expression decreases the tumor’s invasiveness and the ability to form new metastases, thus preventing tumor progression.

In turn, Zhou et al. analyzed the effect of whole pomegranate peel extract. They found that the studied extract, containing 27.58 ± 4.3% polyphenols, selectively inhibited the proliferation of human bladder carcinoma EJ cells [65]. Interestingly, the IC_50_ for EJ cells treated with pomegranate peel extract was 70 μg/mL, while that of the normal rat urinary bladder epithelial cells (RUBE cells) was 200 μg/mL. This selectively observed inhibition of tumor cell proliferation may be a consequence of inducing p53 expression in BC cells by components of pomegranate peel extract, accompanied by the increase of miR-34a expression [65]. A previous study confirmed that p53 protein might specifically activate the miR-34a transcription by binding to responsive elements of miR-34a [66]. In turn, miR-34a may be engaged in suppressing the undue proliferative activity of human BC cells [67]. Thus, a low expression of miR34a was relevant to the malignancy and tumor size in BC patients [68]. Interestingly, in vivo analysis using Balb C nude mice after EJ xenografts revealed a prominently similar relationship as the in vitro study, confirming that pomegranate-induction miR-34a inhibits cancer proliferation through p53 activation [65].

The next in vivo study confirmed the beneficial effect of pomegranate juice in reducing hyperplasia, dysplasia, and invasive neoplasms in the chemically induced (*N*-butyl-*N*-(4-hydroxybutyl)-nitrosamine) BC rat model [69]. Rats with chemically induced BC were characterized by p53 expression impairment, while pomegranate juice treatment restored a regular p53 expression. Therefore, pomegranate juice-induced p53 expression may enable apoptosis activation. In addition to apoptosis, pomegranate juice may reduce oxidative stress. Animals with *N*-butyl-*N*-(4-hydroxybutyl)-nitrosamine-induced BC displayed an increased malondialdehyde level in bladder tissues. In contrast, catalase, glutathione, and superoxide dismutase levels lowered in the cancer-induced group compared to control rats. However, pomegranate juice oral administration restored the normal status of oxidative stress markers [69]. Thus, the anticancer activity of pomegranate juice is also due to its high antioxidant activity [70]. Furthermore, it is noteworthy that previous results confirmed that the antioxidant activity of the whole extract was more effective than its individual components. This can confirm a synergistic action between all biogenic compounds in the pomegranate extract. [71]. Therefore, the use of pomegranate juice is preferable to its ingredients separately. Mortada and colleagues also demonstrated the anti-inflammatory activity of pomegranate juice [69]. Rats with chemically induced BC were characterized by increased expression of *IL-6* (interleukin 6), *TNF-α* (tumor necrosis factor-alpha), and *HIF-1* (hypoxia-inducible factor-1) in bladder tissues [69]. IL-6 and TNF-α are proinflammatory cytokines playing a crucial role in carcinogenesis [72]. A high IL-6 level provides an apoptosis inactivation and induces proliferation of cancer cells, while TNF-α plays a key role in necrosis, invasion, and angiogenesis [72,73]. In turn, a *HIF-1* overexpression carries an increase in the expression of *VEGF* and *bFGF* (basic fibroblast growth factor), which inhibits apoptosis and induces angiogenesis. An in vivo study showed that BC rats, after pomegranate juice administration, showed reduced *IL-6*, *TNF-α*, and *HIF-1* expression compared to BC animals. However, this level remained higher than the normal controls. Therefore, this decreased expression in the BC group after pomegranate juice contributes to angiogenesis suppression and may finally induce cancer cells apoptosis. Thus, pomegranate juice may be effective in BC prevention and treatment [69].

**Figure 1:**
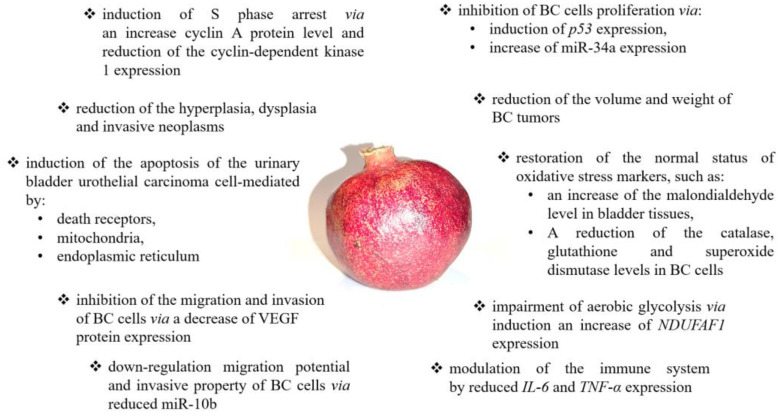
The summary of anticancer effects and mechanisms of pomegranate action. Pomegranate may modulate numerous signaling pathways, including angiogenesis [59], immune response [69], cell proliferation [41], glycolysis [53,54,55], and cell cycle [59,60] as well as apoptosis [23,41,42,47,48,51,53,54,61,62,69,71].

Summarizing the research on the anticancer potential of pomegranate, it should be emphasized that it is impossible to clearly indicate which of the tested products (extracts from the peel, dried plant, fruit extracts, or pomegranate juice) shows the strongest anticancer effect. The comparative analyses to date have concerned only pomegranate peel and fruit extracts, and the obtained results suggest a higher anticancer activity of pomegranate peel extracts. Moreover, pomegranate peel extracts seem more promising in clinical applications because they are characterized by a high selectivity of action only against tumor-altered cells. However, research by Lee et al. and Chang et al. are the only comparative studies [23,41]. Moreover, most of the analyses were performed to focus on different mechanisms of action of the tested extracts, which ultimately determine the anticancer activity of the pomegranate. The studies by Lansky and colleagues are also noteworthy, which indicate a stronger synergistic effect of extracts from the peel of pomegranate fruit than the individual bioactive ingredients of the studied extracts [30]. Nevertheless, there is a justified necessity to conduct further research, which will allow the systemization of the results of research so far. A summary of the anticancer effects and mechanisms of pomegranate action is presented in Figure 1.

## 3. Cranberry

### 3.1. Cranberry—Basic Information

Cranberry (*Vaccinium macrocarpon* Ait. (*Ericaceae*)) is a native plant of North America that is closely related to lowbush blueberry (*Vaccinium macrocarpon* Ait. (*Ericaceae*)), highbush blueberry (*V. myrtillus* L.), bilberry (*V. myrtillus* L.), and lingonberry (*V. vitis-idaea* L.) [74]. Cranberry berries have a pink, red, or dark red color, have a strong sour taste, and may be pear-shaped or ovate, round, oval, flattened, or cylindrical. Cranberry mainly consists of water, organic acids (including salicylate), fructose, vitamin C, flavonoids, anthocyanidins, catechins, and triterpenoids [75]. Base cranberry anthocyanins are galactosides and arabinosides of cyanidin and peonidin [76,77], while approximately 75% of the cranberry flavanols are quercetin glycosides (quercetin 3-*O*-galactoside) [77]. The flavan-3-ols in cranberries are mostly aglycones of catechin and epicatechin. Oligomeric-polymerized tannins composed of successive flavan-3-ol monomeric units also occur in cranberries [77]. A recent study confirmed likewise the presence of some phenolic acid derivatives in cranberries, mainly hydroxycinnamic acid (HCA) and hydroxybenzoic acid (HBA) [77,78]. Iridoid glycosides contained in cranberries are constituents responsible for their taste. Moreover, vaccinium berries contain anthocyanidins and proanthocyanidins (PAC), responsible for the defense against microbes [75,79]. Previous studies have confirmed that the variety of compounds in cranberries has many health benefits, such as protection against lipoprotein oxidation, prevention of bacterial adhesion in urinary tract infections (UTIs) of *Escherichia coli* and stomach ulcers, and in vitro anticancer activity. Moreover, flavonoids and anthocyanins from the extracts of cranberry fruits have been identified as significant antioxidants [74].

In addition to the many proven beneficial effects of cranberries, its excessive consumption may adversely affect the body, especially in people taking medications. In vitro and in vivo studies confirm that, like grapefruit juice, cranberry juice can inhibit cytochromes P450 (CYP3A4 and CYP2C9 enzymes), and a glycoprotein P transporter is related to drug metabolism. P-glycoprotein is expressed in epithelial cells related to the drug’s absorption and distribution, e.g., in the tubular membrane of hepatocytes, proximal renal tubules, intestinal mucosa, and brain capillaries [80]. Thus, the knowledge of the drug-cranberry interaction starts, especially in the case of therapy with warfarin (an anticoagulant drug, the so-called vitamin K antagonist). Consumption of large amounts of cranberry juice (1–2 liters per day) or concentrate (1000 mg per day) for a long time (>3–4 weeks) can be correlated with changed INR values [81]. In addition, consuming cranberry juice is not recommended for people with urolithiasis. Consuming large amounts of cranberry juice (1 L/day) reduces urinary pH through decreased urinary uric acid, retarding urate synthesis. Therefore, cranberry juice intake increases the risk of calcium oxalate and uric acid stone formation but decreases the risk of brushite stones [82].

### 3.2. Cranberry in Bladder Cancer Prevention

A previous study confirmed that cranberry juice shows potential beneficial effects in UTI prevention. Howell and Foxman found that a cranberry juice cocktail prevented the adhesion of 80% of non-resistant isolates of fimbriated *E. coli* strains and 79% of antibiotic-resistant isolates in vitro [83]. Subsequent studies indicated the potential mechanism of action of cranberry on the bacteria, causing UTIs. UTIs are caused mainly by *E. coli* bacteria, whose pathogenicity is primarily due to the ability of fimbriae to adhere to the urinary epithelium cells. The cranberry hypothesis assumes that its components—proanthocyanidins and fructose—inhibit the adherence of fimbria to epithelial cells, thus preventing infection [84]. Due to the promising results of in vitro studies, in vivo studies were undertaken. However, clinical trials have shown that cranberry juice is ineffective in treating UTIs [79,85]. On the other hand, several studies have confirmed the effectiveness of cranberry in the prevention of UTIs, especially with recurrent infections, including cystitis [86]. The ability of cranberry extracts to prevent urinary tract infections can be used as an adjunct to radical pelvic radiotherapy in patients with bladder and cervical cancer. In addition to frequent urination, cystitis is the most common side effect of radical pelvic radiotherapy. Twice daily (morning and evening) use of cranberry juice in patients during radiotherapy and for two weeks after treatment (6 weeks total) resulted in a reduction in the incidence of severe urinary symptoms or urinary tract infections (82.5%) compared to the control group (89.3%) [87].

Positive results in the use of cranberries in the prevention of UITs, including cystitis, contributed to further research on extending the prophylactic use of cranberries in urinary tract cancers. The main ingredients of cranberries are flavonoids, including quercetin. An in vitro study, including a panel of human bladder tumor cell lines (RT4, SCABER, and SW-780) and non-tumorigenic immortalized human uroepithelial cells (SV-HUCs), showed that quercetin 3-*O*-glucoside, isorhamnetin (3′-*O*-methylquercetin), myricetin, and quercetin were characterized by strong concentration-dependent cell growth inhibitory activities in BC cells, with IC_50_ values in a range of 8–92 μM [88]. Interestingly, isorhamnetin and myricetin showed deficient inhibitory activity against non-cancerous SV-HUC cells even at very high concentrations (>200 μM) compared with BC cells, indicating their cytotoxicity is selective for cancer cells. This selective cytotoxicity gives these compounds a potent therapeutic and preventive potential for BC [88]. On the other hand, the anticancer properties of cranberry may be a result of impaired angiogenesis by inhibition of VEGF-related vascularization, therefore preventing tumor growth [89]. Roy and colleagues found that cranberry extract significantly inhibited both H_2_O_2_ as well as TNF-induced VEGF expression in vitro [89]. However, these studies were performed on the human keratinocytes but not on bladder cells; therefore, further studies are needed [89].

**Figure 2:**
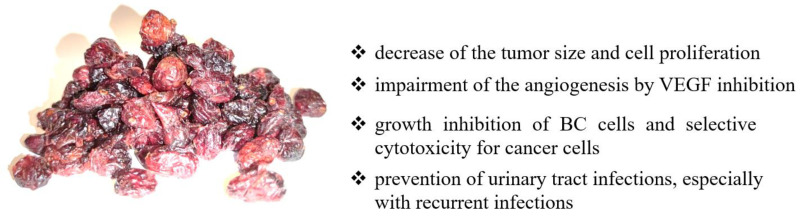
The summary of anticancer effects and mechanisms of cranberry action. Cranberry may modulate numerous signaling pathways, including angiogenesis [89], growth and proliferation [89], and inflammation [79,83,84,85,86,87].

The chemopreventive efficacy of cranberry juice concentrate has been confirmed in an animal study of Fischer-344 female rats with chemically induced (using *N*-butyl-*N*-(4-hydroxybutyl)-nitrosamine) urinary BCs. The animals were administered juice in two doses of 1.0 or 0.5 mL/rat/day, beginning one week after the final *N*-butyl-*N*-(4-hydroxybutyl)-nitrosamine treatment and continuing for six months (until the end of the study). LC-MS and MS/MS analysis confirmed that the cranberry juice concentrate contained the main flavonoids and anthocyanins, existing in conjugated form with various sugars (pentose and hexoxides). These conjugated forms significantly influence their bioavailability and absorption. The cranberry treatment at dose 1.0 mL/rat/day caused a 38% reduction in the cancer numbers compared to the control group that received water, while 0.5 mL/day dose caused a non-significant decrease of only 7%. Moreover, cranberry concentrate therapy led to a reduction in the weight of the bladders by 31% at the high dose and 5% at the low dose, indicating a decrease in tumor size and suggesting a decreased cell proliferation. Thus, these results suggest that cranberry juice concentrate may be inhibiting urinary BCs by blocking cell proliferation [90]. Additional LC-MS/MS analysis of plasma and urine showed that quercetin and its methylated derivative, as main flavonolignans of cranberry juice, were detected in the urine samples while not reported in the serum samples, indicating its poor bioavailability. On the other hand, the highest level of cranberry components in urine compared to other body fluids indicates their selective action on urothelial cells [90]. A summary of anticancer effects and mechanisms of cranberry action is presented in Figure 2.

## 4. Citrus

### 4.1. Citrus—Basic Information

Citrus fruits (*Rutaceae* family) with 100 million tons of production per season comprise the largest fruit sector worldwide. Citrus fruits such as oranges, lemons, grapefruits, pomelos, and limes are widely grown [91]. Citrus fruits contain many valuable components, such as sugars, dietary fiber, folic acid, potassium, magnesium, copper, calcium, niacin, vitamin B6, thiamine, phosphorus, riboflavin, pantothenic acid, and ascorbic acid (vitamin C) [92]. Moreover, citrus fruits are a rich source of phytochemicals that do not necessarily serve the survival of plants but represent pharmacological activity. The phytochemicals found in citrus are flavonoids, alkaloids, coumarins, limonoids, carotenoids, phenol acids, and essential oils. These active secondary metabolites show several health benefits, including antioxidative, anti-inflammatory, anticancer, cardiovascular protective effects, and neuroprotective effects [93]. Interestingly, citrus has been used as medicine in China, Japan, and Korea. Nine traditional Chinese medicines containing six species of citrus (*C. reticulata* Blanco, *C. medica* L. var. *sarcodactylis Swingle*, *C. medica* L., *C. wilsonii* Tanaka, *Citrus aurantium* L., and *C. sinensis* (L.) Osbeck) have been registered in the Chinese Pharmacopoeia for appropriate medical use [93].

As with the consumption of pomegranate and cranberry, the consumption of citrus is associated with no side effects. However, citrus may alter the pharmacokinetics of used drugs. The effect of citrus (mainly grapefruit juice) is related to the reduction of intestinal CYP3A4 activity as well as the inhibition of the P-glycoprotein transporter. Thus, citrus juice may increase the oral bioavailability of drugs, e.g., cyclosporin, that are P-glycoprotein and CYP3A4 substrates [80].

### 4.2. Citrus in Bladder Cancer Prevention

Due to its potential anticancer properties, *Citrus unshiu* Markovich (satsuma citrus), which belongs to the *Rutaceae* family, has been very popular in recent decades. It is a thin-peel citrus originating from Satsuma city (now Kagoshima) on Kyushu island in Japan. Ahn and colleagues showed that ethanol extract of *C. unshiu* Markovich peel inhibited the growth of human BC T24 cells [94]. The antitumor activity of the studied extract is based on its ability to increase ROS production and the inactivation of the ROS-dependent PI3K/Akt pathway in T24 cells, which contributes to apoptosis induction. Subsequent studies confirmed that T24 cells showed an increase in caspase-8 and -9 activation after exposure to *C. unshiu* Markovich extract. The extrinsic pathway is mediated by caspase-8, whereas the intrinsic pathway can be activated by caspase-9. Thus, this elevated activation of both caspases suggests that both external and internal pathways might induce apoptosis. Moreover, *C. unshiu* Markovich-treated T24 cells also demonstrated an enhanced expression of death-receptor-related proteins, the loss of mitochondrial membrane integrity, and the increased translocation of Cyt c from mitochondria to the cytoplasm, as well as an increased expression of Bax. On the other hand, T24 cells after *C. unshiu* Markovich treatment were characterized by the reduced expression of BCL2 and IAP (inhibitor of apoptosis) family proteins (XIAP, cIAP-1, and cIAP2) [94]. BCL2 is a typical antiapoptotic protein that suppresses this process [95,96], while IAPs selectively bind to caspases and block apoptosis [97,98]. Subsequent molecular analyses also confirmed the increased caspase-3 activity and PARP (poly-(ADP-ribose)-polymerase-1) cleavage in T24 cells treated with the studied extract. Moreover, increased caspase-8 expression may contribute to the truncation of Bid (BH3 interacting-domain death agonist). In turn, truncated Bid acts as a linker molecule linking death receptor and mitochondrial-dependent pathways [94]. Thus, *C. unshiu* Markovich extract caused an increase of truncated Bid level, which facilitated caspase-9 activation and, finally, cell death [99,100]. Summarizing the induction of apoptosis results, it should be emphasized that the studied extract induced activation of both intrinsic and extrinsic apoptosis pathways, wherein the extrinsic pathway eventually amplified the intrinsic pathway through the caspase-8-mediated truncation of Bid. Moreover, the extract from *C. unshiu* Markovich also inhibited PI3K and Akt phosphorylation, enhancing apoptosis induction [94].

The next in vitro study focused on the anticancer properties of modified citrus pectin (MCP). MCP was obtained from the peel and flesh of citrus fruit and modified into a complex, water-soluble, indigestible polysaccharide, processed at high temperature and pH [101]. Previous studies showed that the main target of MCP is galectin-3, whose overexpression has been reported in multiple cancer types, including breast, lung, gastric, liver, and bladder cancers [102,103,104,105]. Galectin-3 is an oncogenic protein that is involved in numerous processes, including cell growth, differentiation and adhesion, RNA splicing, apoptosis, neoplastic transformation, and metastasis, as well as being the guardian of the tumor microenvironment, suppressing immune surveillance by killing T-cells and interfering with NK cell function [106,107,108]. Thus, previous results suggest that galectin-3 overexpression is related to high tumor grade and poor survival and is a potential therapeutic target in human BC patients [109]. Fang et al. found that MCP may inhibit tumor growth via induction of cell cycle arrest and apoptosis in BC cells in vitro and in vivo [109]. MCP induced G_2_/M phase arrest through the downregulation of cyclin B1 and the phosphorylation of cyclin-dependent kinase 1 (Cdc2). Moreover, both T24 and J82 cells were characterized by a two-fold increase in caspase-3 activity and exhibited fragmented nuclei, the main characteristic of apoptosis. Proteome analysis reported that MCP reduced galectin-3 protein expression. Moreover, MCP-treated BC cells showed reduced Akt phosphorylation and the associated low phosphorylation of the p-Bad and p-S6 ribosomal protein. p-Bad is phosphorylated by p-Akt to promote cell survival, while p-S6 ribosomal protein is phosphorylated by p-Akt to facilitate protein synthesis in cell cycle progression. However, MCP showed no impact on MAPK (mitogen-activated protein kinase) signaling, confirming the unchanged level of p-Erk1/2. Importantly, in vitro studies have been confirmed by in vivo studies. After palpable tumor development, nude mice injected subcutaneously with T24 cells were treated with MCP (two doses of 350 and 700 mg/kg MCP). Analyses showed that the animals receiving the highest dose of MCP had the lowest tumor growth and the lowest mass of the excised tumor. According to the in vitro results, the expression level of galectin-3 protein was also decreased MCP-treated T24 xenografts. Moreover, Fang et al. showed that the highest-dose MCP treatment caused a decrease of the proliferative index by 68%, accompanied by a remarkable increase of the apoptosis index by 30%, as compared to the control group that received a vehicle (RPMI 1640 medium) [109]. These results confirmed that MCP inhibits carcinogenesis via galectin-3 inactivation and causes cycle arrest and apoptosis. Therefore, galectin-3 may be the potential target of cancer therapy if we consider citrus extract as a preventive and therapeutic agent for bladder cancer. 

Another significant compound commonly found in grapefruit and other citrus fruits is naringin. It is an active flavonoid that has an antioxidant [110] and antiatherosclerotic [111] effect, as well as antiviral activity [112], and it lowers the level of the cytochrome P450 1A2 protein [113]. Moreover, previous studies have found that an increased naringin intake is associated with a decreased risk of various cancers, including breast and lung cancer [114,115]. Kim and colleagues found that naringin may induce a cell growth inhibition due to p21WAF1-mediated G_1_-phase cell-cycle arrest via the Ras/Raf/Erk-signaling pathway in cancer cells [116]. Interestingly, naringin-treated 5637 cells were characterized by selectively decreased viability and thymidine uptake, while this effect was not observed in the case of normal fibroblast cells. Molecular analysis confirmed that naringin may cause cell cycle arrest in the G_1_ phase, which may be accompanied by a decrease in cyclin D1/cyclin-dependent kinase 4 (CDK4) and cyclin E/CDK2, involved in the progression of the cell cycle from G_1_ to S phase. Moreover, upregulation of p21WAF1 was observed during the G_1_-phase arrest in 5637 naringin-treated cells. However, naringin did not affect p27 and p53 expression, suggesting that naringin-induced p21WAF1 accumulation is independent of the p53 pathway. Moreover, naringin treatment led to the upregulation of Erk, JNK, and p38 MAPK phosphorylation [116]. The MAPK pathway plays a crucial role in inhibiting cell growth [117] and/or regulating the cell cycle [118,119]. Erk pathway stimulation by naringin may contribute to the induction of cell-cycle inhibitor proteins, e.g., p21WAF1, causing a suppressed cell-cycle progression. On the other hand, naringin may also induce the inhibition of cell growth by activating Ras without affecting the level of expression of this protein. The activated Ras then activates Erk and an increase in p21WAF1 expression, inhibiting the expression of CDK2 and CDK4, with the consequent arrest of the G_1_ phase cell cycle [116].

**Figure 3:**
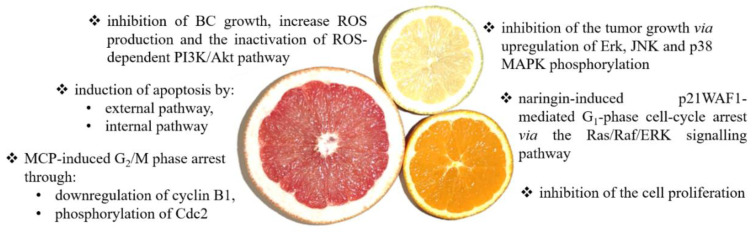
The summary of the anticancer effects and mechanisms of citrus action. Citrus fruit may modulate numerous signaling pathways, including apoptosis [94,95,96,97,98,99,100], ROS generation [94], growth and proliferation inhibition [109], and cell cycle [109,116].

It is notable that a clinical study showed that in the case of patients who consumed citrus 3–4 times/week and daily, the cancer risk was reduced by 11% (RR = 0.89, 95% CI = 0.80–0.9) and 17% (RR = 0.83, 95% CI = 0.73–0.93), respectively. However, in the case of bladder cancer, the obtained results were not statistically significant [119]. Unfortunately, the results of the epidemiological studies to date regarding the impact of citrus fruit consumption on the reduction of the risk of bladder development remain inconclusive [120]. Accordingly, Liang et al. performed a meta-analysis that included eight case–control studies and six cohort studies [121]. There was a significant inverse association between the intake of citrus fruit and BC risk in all pooled studies (RR: 0.85; 95% CI, 0.76–0.94) and case–control studies (RR: 0.77; 95% CI, 0.64–0.92) but not in the cohort studies (RR: 0.96; 95% CI, 0.87–1.07). Nevertheless, the meta-analysis shows that the consumption of citrus significantly reduces the risk of developing multiple BCs [121]. Moreover, the multi-ethnic cohort study showed that citrus fruit intake was inversely associated with the risk of invasive BC in women (HR = 0.56, 95% CI = 0.34–0.90) [11,118]. A systematic review and meta-analysis of eight prospective studies, being a part of the *World Cancer Research/American Institute for Cancer Research Continuous Update Project*, aimed to assess the dose–response relationship between fruit and vegetables and incidence and mortality of BC. Among studied fruits, citrus was the most effective in reducing BC risk (RR = 0.87, 95% CI = 0.76–0.99) [122]. Analysis of modifiable risk factors for BC prevention showed that fruits (RR = 0.77, 95% CI = 0.69–0.87), vegetables (RR = 0.83, 95% CI = 0.75–0.92), citrus fruits (RR = 0.85, 95% CI = 0.76–0.94), and cruciferous vegetables (RR = 0.84, 95% CI = 0.77–0.91) may contribute to reduction of the BC risk [123]. A protective effect against BC development with citrus fruit consumption was also noted (pooled RR = 0.83, 95% CI = 0.69–1.01) in East Asians [124]. Summing up, the conducted meta-analyses confirm the beneficial effect of citrus consumption in reducing the risk of bladder cancer development. A summary of anticancer effects and mechanisms of citrus action is presented in Figure 3.

## 5. Cactus Pear

### 5.1. Cactus Pear—Basic Information

Cactus pear is a rich source of pectin, carotene, betalains, ascorbic acid, quercetin, and quercetin derivatives, all of which have antioxidant activity [125]. In Chinese medicine, cactus pear is considered a weak venom used to treat inflammation and pain and a detoxifying agent for snake bites [126].

Nevertheless, cactus pear extracts should be used with caution, especially those with hyperglycemia. Previous studies have shown that the cactus pear effectively lowers blood sugar levels. Therefore, the concomitant use of antihyperglycemic drugs and herbal remedies containing cactus grass may lead to hypoglycemia [127].

### 5.2. Cactus Pear in Bladder Cancer Prevention

**Figure 4:**
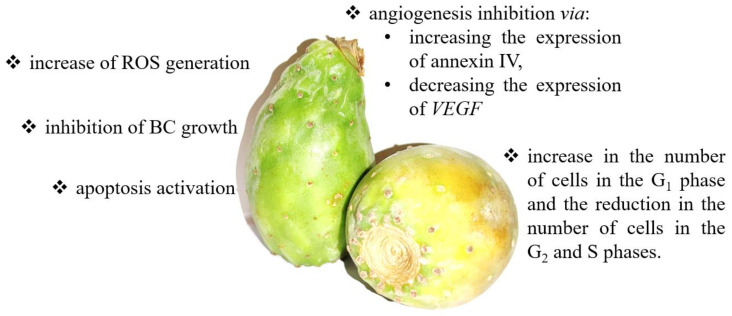
The summary of the anticancer effects and mechanisms of cactus pear action. Cactus pear may modulate numerous signaling pathways, including apoptosis [129], cell growth [128], angiogenesis [128], cell cycle [128], and ROS generation [129].

Since cactus pears are a rich source of antioxidants, they also appear to have anticancer properties. Zou et al. reported that an aqueous cactus pear extract inhibited the growth of T24 cells [128]. After cactus pear extract treatment, an elevated apoptosis activation was observed and an increased cell number in the G1 phase and decreasing cell number in the G_2_ and S phases in vitro. Moreover, molecular analysis showed that exposure to cactus pear extract enhanced the expression of annexin IV and reduced VEGF levels in neoplastic cells. Annexin IV is part of the annexin family of calcium-dependent phospholipid-binding proteins, which regulate cell proliferation, apoptosis, and tumor progression. In turn, as mentioned above, VEGF is primarily involved in the regulation of angiogenesis during tumor progression [128]. Thus, blocking these two factors effectively reduces tumor growth, influencing the formation of new blood vessels and the migration of tumor cells. Another in vitro study showed that cactus pear extract amplified oxidative stress by increasing ROS and p16 and RASSF-1A DNA methylation in BC cells. P16 is a cell cycle regulator that controls the G_1_ phase of the cell cycle to the S phase via the inhibition of CDK4 and CDK6. RASSF-1A is a tumor suppressor involved in regulating the cell cycle, apoptosis, and microtubule stability through the regulation of Ras signaling. Therefore, the higher methylation status of p21 and RASSF-1A contributes to reducing their expression and finally leads to cell cycle arrest and apoptosis induction. RASSF-1A can inhibit the accumulation of cyclin D1 and thus induce cell cycle arrest. At the same time, p21 is a potent inhibitor of cyclin-dependent kinases that directly inhibit the activity of the complexes, cyclin E/CDK2, and cyclin D/CDK4. Blocking these complexes prevents the cell from entering the S phase of the cell cycle [129,130]. A summary of anticancer effects and mechanisms of cactus pear action is presented in Figure 4.

## 6. Apple

### 6.1. Apple—Basic Information

Apples are a rich source of polyphenols, including chlorogenic acid, p-coumaroylquinic acid, catechin, epicatechin, procyanidins, quercetin-3-*O*-rutoside, phloretin, floridine, and anthocyanins (cyanidin-3-*O*-galactoside), which show antioxidant, anti-inflammatory, and anticancer properties [131].

Additionally, in the case of apples, possible adverse interactions with oral drugs are observed. Due to the presence of inhibitors, organic anion-transporting polypeptides (OATPs) in apple juice inhibit the absorption of drugs in the gastrointestinal tract. OATPs are a group of membrane transport proteins that facilitate the influx of endogenous and exogenous substances through biological membranes. OATPs are found in enterocytes and hepatocytes and the brain and kidneys. Consequently, apple juice reduces the gastrointestinal absorption of some antiallergic drugs, antibiotics, antihypertensive drugs and beta-blockers, and even psychotropic drugs. Therefore, these patients should take their medication at least 4 hours prior to fruit juice consumption [132].

### 6.2. Apple in Bladder Cancer Prevention

The antioxidant and anti-inflammatory properties of the compounds present in apples may also help reduce BC’s development. Kao and colleagues showed that Applephenon^®^ (AP) might be a potential chemopreventive agent against BC [130,133]. AP is a commercial product purchased from Asahi Co., Tokyo, Japan, prepared from unripe apples (*Malus pumila* cv. *Fuji*) [134]. Human urinary bladder cancer cells (TSGH-8301) exposed to AP for 24 hours showed morphological changes characteristic of apoptosis, such as membrane blebbing and nuclear condensation [133]. The induced apoptosis in AP-treated cells is a consequence of the activation of the mitochondria-dependent pathway. AP exposure resulted in the downregulation of BCL2, upregulation of apoptosis-inducing factor (AIF), and activation of caspase-3. AIF is a mitochondrial protein that activates apoptosis in a caspase-independent pathway by inducing chromatin condensation and DNA fragmentation. It is usually located behind the outer mitochondrial membrane and therefore separated from the nucleus. However, when the mitochondria are damaged, it migrates into the cytosol and nucleus [135]. Moreover, a reduction in mitochondrial potential was observed in cells. Similarly, an in vivo study using mice xenografted with TSGH-8301 confirmed a reduction in BCL2 expression after AP treatment.

On the other hand, AP showed antiproliferation potential. A 24-h exposure of TSGH-8301 cells to AP resulted in a significant accumulation of cells in the G_2_/M phase and a low in cells with G_0_/G_1_ phase accompanied by a decreased Cdc2and cyclin B [133]. Cdc2-cyclin B, also known as CDK1-cyclin B (cyclin-dependent kinase 1), is an element of the cyclin-dependent kinases complex involved in the cell cycle control; it drives the onset of mitosis. During the G_2_ phase, the Cdc2-cyclin B complex is kept inactive by the phosphorylation of Cdc2 by CDK1 inhibitory protein kinases. Until the late phase of G_2_, dephosphorylation by protein phosphatase of the cell division cycle (Cdc25) activates the Cdc2-cyclin B1 complex and triggers the initiation of mitosis [136]. 

Interestingly AP-treated TSGH-8301 cells were characterized by micronucleation and multinucleation as well as a decreased protein level of α-tubulin. Therefore, these results suggest that AP may induce mitotic catastrophe. Importantly, BC cells after AP treatment showed reduced ROS generation. However, the reason for this is still unclear, whether the reduction in ROS is due to a decrease in cell numbers or a change in the regulation of internal molecules in a BC cell [133].

An animal BC model (in female Fischer 344 rats, BC was induced by administration of *N*-butyl-*N*-(4-hydroxybutyl) nitrosamine in the drinking water for ten weeks) confirmed that AP could decrease the tumor progression. Moreover, analysis of tumor specimens from the AP-treated animals showed reduced expression of BCL2, cyclin B1, and PCNA (proliferating cell nuclear antigen) and increased Bax and Cip1/p21 expression [130]. Thus, AP decreased cell proliferation accompanied by a suppression of tumor growth in the bladder, with the reduced cell proliferation due to the cell arrest in the G_2_/M phase of the cell cycle [130]. On the other hand, AP may regulate the cell cycle via modulation of p21 expression. AP may also limit the proliferation and growth of neoplastic cells by reducing the expression of PCNA. PCNA is a protein involved in the replication process, helping to increase the processivity of the lead-strand DNA synthesis. In turn, in response to DNA damage, this protein undergoes ubiquitination and participates in the RAD6-dependent DNA repair pathway [137].

On the other hand, AP can induce apoptosis via reduction of BCL2 protein levels and an increase in the expression level of Bax and Cip1/p21, as well as the level of cleaved PARP in the bladder urothelium. Molecular analysis showed that AP-treated rats were characterized by a decreased NF-κB level, accompanied by a simultaneous reduction in *HO-1* (heme oxygenase 1) expression compared to rats treated only with *N*-butyl-*N*-(4-hydroxybutyl)-nitrosamine. HO-1 deficiency in normal cells can enhance DNA damage and carcinogenesis, while in cancer cells, *HO-1* overexpression could promote proliferation and survival [138]. In turn, NF-κB is a transcription factor activated by various growth factors and cytokines from the stromal cell and can induce *HO-1* gene transcription in tumor cells. Summarizing the above data, apple-derived AP has potential as an anticancer agent against human BC due to the ability to block the cell cycle and induce apoptosis.

**Figure 5:**
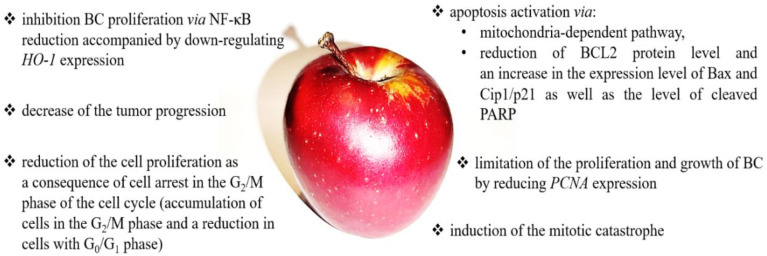
The summary of the anticancer effects and mechanisms of apple action. Apple may modulate numerous signaling pathways, including apoptosis [131,133,135,138], proliferation and cell growth [131,133,137,138], cell cycle [131,133,136], and mitotic catastrophe [131].

Interestingly, a clinical study showed that hard fruit (pear and apple) consumption, with increments of 25 g/day, was correlated with a 7% protective association on BC risk among non-smokers (HR = 0.93, 95% CI= 0.89–0.98) [139]. Sacerdote et al. confirmed the results of previous in vitro studies and reported that apple intake above-median contributed to reduced BC risk (OR 0.63, CI 95% = 0.39–0.99) [140]. A summary of anticancer effects and mechanisms of apple action is presented in Figure 5.

## 7. Conclusions

Considering the variety of compounds present in the fruits in question, apples, cactus pears, pomegranates, cranberries, and citrus may effectively reduce the risk of BC development. The studied fruits are a rich source of phenols, flavonoids, ellagitannins, tannins, anthocyanidins, catechin, epicatechin, and quercetin. Although it is clear that the described fruits have beneficial health-promoting effects, including antitumor effects, most of the studies have been carried out in cell cultures and animal models. Therefore, despite the promising results of the research to date, it is necessary to conduct extensive clinical trials to verify the hypotheses. Moreover, detailed clinical studies are required to examine potential side effects of consumed fruits, especially their interactions with orally administered drugs.

Nevertheless, the obtained results indicate two main molecular mechanisms that determine the anticancer properties of the analyzed fruits. Firstly, the studied fruits are able to arrest the cell cycle, thus preventing cell proliferation and tumor progression. Secondly, the compounds present in the fruit induce apoptosis through all possible pathways. The regulation of these two pathways guarantees the inhibition of the development and progression of the pattern. However, it should be remembered that the existing hypotheses have been developed primarily on the basis of in vitro studies and animal studies; therefore, they should be verified with clinical trials.

## Data Availability

No new data were created or analyzed in this study. Data sharing is not applicable to this article.
